# The Physiological and Performance Effects of Actovegin during Maximal Cardiopulmonary Exercise Testing: A Randomized, Double-Blind, Placebo-Controlled Trial

**DOI:** 10.3390/nu16193332

**Published:** 2024-09-30

**Authors:** Dragana Milovanović, Dragan Radovanović, Vladimir Živković, Ivan Srejović, Miloš Glišić, Vladimir Jakovljević, Aaron Scanlan, Nenad Ponorac, Emilija Stojanović

**Affiliations:** 1 Military Medical Academy, 11000 Belgrade, Serbia; dramilovanovic@gmail.com; 2 Faculty of Sport and Physical Education, University of Niš, 18000 Niš, Serbia; fiziologija@fsfv.ni.ac.rs; 3 Faculty of Medical Sciences, Department of Physiology, University of Kragujevac, 34000 Kragujevac, Serbia; vladimirziv@gmail.com (V.Ž.); ivan_srejovic@hotmail.com (I.S.); miloskg92@gmail.com (M.G.); drvladakgbg@yahoo.com (V.J.); 4 Department of Human Pathology, 1st Moscow State Medical University IM Sechenov, 119991 Moscow, Russia; 5 School of Health, Medical and Applied Sciences, Central Queensland University, Rockhampton 4701, Australia; a.scanlan@cqu.edu.au; 6 Faculty of Medicine, Department of Physiology, University of Banja Luka, 78101 Banja Luka, Bosnia and Herzegovina; nenad.ponorac@med.unibl.org; 7 Department of Training and Exercise Science, Faculty of Sport Science, Ruhr University Bochum, 44801 Bochum, Germany

**Keywords:** VO_2max_, calf blood, anti-doping, aerobic capacity, Solcoseryl, cardiorespiratory fitness

## Abstract

Background: Evidence regarding the performance-related effects of Actovegin is limited, despite legislated restrictions being in place for this supplement within sport settings. Objectives: Our study examined the effects of Actovegin on physiological responses and performance during maximal cardiopulmonary exercise in collegiate athletes. Methods: A randomized, double-blind, placebo-controlled experimental design was adopted. Moderately trained collegiate athletes from various sports were randomly allocated to placebo (*n* = 8) or Actovegin (*n* = 8) groups. All athletes consumed three capsules across each day for seven days of loading. Athletes underwent two separate cardiopulmonary exercise tests one week apart. Separate 2 × 2 mixed ANOVAs and effect sizes ($\eta_{p}^{2}$) were used to assess for between- and within-group differences. Results: A significant time * group effect (*p* = 0.036, $\eta_{p}^{2}$ = 0.278) was observed in systolic blood pressure. Significant main effects were only observed for time in several variables, with increases in peak oxygen uptake (VO_2_) (*p* < 0.001, $\eta_{p}^{2}$ = 0.893), peak minute ventilation (*p* = 0.004, $\eta_{p}^{2}$ = 0.456), ventilatory equivalents for carbon dioxide (*p* = 0.002, $\eta_{p}^{2}$ = 0.517), oxygen pulse (*p* = 0.006, $\eta_{p}^{2}$ = 0.434), VO_2_ at first ventilatory threshold (*p* = 0.002, $\eta_{p}^{2}$ = 0.520), velocity at second ventilatory threshold (*p* < 0.001, $\eta_{p}^{2}$ = 0.997), VO_2_ at second ventilatory threshold (*p* < 0.001, $\eta_{p}^{2}$ = 0.628), and peak velocity (*p* = 0.010, $\eta_{p}^{2}$ = 0.386), and a decrease in respiratory exchange ratio (*p* < 0.001, $\eta_{p}^{2}$ = 0.695). Conclusions: Our findings suggest that although physiological and performance alterations were evident with Actovegin supplementation during cardiopulmonary exercise, no further benefits beyond those obtained with a placebo were attained.

## 1. Introduction

Actovegin (or Solcoseryl) is a deproteinized hemoderivative of calf’s blood composed of more than 200 bioactive ingredients. The main ingredients are low molecular weight substances, including amino acids, sphingolipids, lactate, succinate, vitamins, adenosine monophosphate, and inositol-phospho-oligosaccharide [1]. Several of these bioactive ingredients affect various biochemical pathways to combat a range of medical conditions, including ischemic stroke and brain injury [2], peripheral arterial and venous perfusion disorders [3], diabetic polyneuropathy [4], and skin trauma conditions [1]. Concerning mechanisms of action, Actovegin: (i) modulates the nuclear factor kappa B, a protein complex which regulates inflammatory and apoptosis processes; (ii) inhibits excessive poly (ADP-ribose) polymerases activation, which underpins morphological and functional improvements in the peripheral and central nervous systems; and (iii) promotes anti-hypoxic effects by improving the absorption and utilization of oxygen, which inhibits the production of lactate and stabilizes cellular plasma membranes during ischemia [5]. Although Actovegin is frequently administered in clinical practice to treat different medical conditions, or in animals with potential translation of results on athletic performances [6,7], evidence supporting its use in sport settings in humans are still scarce.

Actovegin has been proposed to hold hypoalgesic effects in treating muscle injuries by countering disturbances to muscle blood flow and energy imbalances via modulation of inflammatory processes and ischemia [8,9]. However, despite the potential clinical application of Actovegin in sport, it has only been investigated in one study within sport settings [8]. Specifically, Lee et al. [8] demonstrated faster recovery from hamstring tears in professional soccer players receiving Actovegin injections at a dose of 2 mL (40 mg·mL^−1^), allowing an 8-day earlier return to play compared to controls who only underwent a physiotherapy protocol. Despite Actovegin being documented to safely treat muscle injuries [8], some concerns have arisen regarding its use in sport. In this regard, an anaphylactic reaction with multi-organ failure was reported in a 22-year-old amateur cyclist after intravenous administration of Actovegin at a dose of 5 mL (40 mg·mL^−1^) [10]. In turn, several sources highlight injectable [9,11] and oral [9,12,13,14] Actovegin abuse has been prevalent in endurance-based sports over the past three decades [9,11,12,13,14,15]. In this regard, Actovegin was added to the list of banned substances (under the category of blood doping agents) by the International Olympic Committee in 2000, but it was removed soon after due to insufficient evidence demonstrating an ergogenic effect. Nevertheless, the current World Anti-Doping Code stipulates that intravenous injections of Actovegin exceeding 100 mL are prohibited and further serial injections must be at least 12 h apart, with no restrictions placed on dosages that are administered orally. Effective detection of ever-evolving prohibited performance-enhancing substances and practices are essential in refining anti-doping regulations and safeguarding doping-free sport competition, especially considering the frequency of drug misuse in sport and increased availability of illegal substances on the black market [16].

The established regulations for Actovegin use have been instilled despite evidence on the performance-related effects for this supplement being limited. Indeed, the lack of empirical attention towards Actovegin might stem from ethical concerns given the controversial history and legislative restrictions associated with its use. However, from a mechanistic standpoint in supporting the potential ergogenic effects of Actovegin in endurance sports, Søndergård et al. [17] identified a dose-dependent (10 μL·mL^−1^ and 50 μL·mL^−1^) increase in mitochondrial oxidative phosphorylation capacity, rates of mitochondrial adenosine triphosphate production, and adenosine diphosphate sensitivity of skeletal muscle with Actovegin treatment. It has been postulated that the insulin-like activity and enhanced oxygen supply properties thought to accompany Actovegin supplementation may underpin its potential to elicit aerobic-oriented performance-enhancing outcomes in sport settings by promoting anti-hypoxic effects [9]. Although these improvements may reflect an enhanced oxidative capacity, data were collected in vitro in permeabilized skeletal muscle among untrained, overweight subjects, so it remains to be confirmed if these findings translate to benefits in endurance performance among athletes. Although the use of Actovegin has been anecdotally popularized for its performance-enhancing potential among endurance athletes, only one study has surprisingly been conducted on this topic in the literature [18]. Lee et al. [18] directly reported no significant benefits of intravenous Actovegin (40 mL) administration on various metabolic and performance variables during a maximal, graded arm crank ergometry test in physically active men (*n* = 8). While useful, these findings are limited to a small sample of active subjects as opposed to athletes and are restricted to an unfamiliar upper-body exercise rather than a more common exercise, such as running. Consequently, the limited available evidence concerning the ergogenic effects of Actovegin in athletes emphasize a need for further research in this area, especially given the restrictions placed on its use in sport. In this regard, our placebo-controlled, double-blind study aimed to examine the physiological and performance effects of Actovegin during maximal cardiopulmonary exercise in collegiate athletes. We hypothesized that the oxidative-enhancing capacity of Actovegin reported in in vitro research [17] may underpin benefits to physiological and performance variables during maximal cardiopulmonary exercise beyond those attained with a placebo.

## 2. Methods

### 2.1. Subjects

A sample of 14 male and 10 female, moderately trained collegiate athletes (Tier 2 in the Participant Classification Framework) [19] were initially recruited and screened for participation in this study. Screening procedures included completion of a questionnaire to provide information on health history, menstrual status, potential banned substance use, and training history, as well as completion of a maximal cardiopulmonary exercise test. Inclusion criteria for participation in the study were: (1) 18–25 years of age; (2) possessing at least 3 years of training and competition experience immediately prior to the study, performing ≥5 h·week^−1^ of exercise in any sport; (3) compliance with the list of substances and methods banned by the World Anti-Doping Agency; (4) free from injury/illness at the time of the study; (5) no history of cardiovascular disorders, diabetes mellitus, arterial hypertension, or dyslipidemia; (6) regular menstrual cycle prior to participation in the study; and (7) a peak oxygen uptake (VO_2peak_) attained during the test classified as fair or good according to sex- and age-stratified guidelines established by the American College of Sports Medicine [20]. After an initial screening, 20 subjects were included in the study with 1 male and 3 females excluded due to having a history of cardiovascular disorders (*n* = 1) or an insufficient VO_2peak_ (*n* = 3). In addition, 3 males and 1 female experienced injury or illness across the study period and were thus excluded. Therefore, a total of 16 subjects completed the study. The study was approved by the Ethics Committee of the Faculty of Medical Sciences, University of Kragujevac (01-7002). All subjects provided informed consent prior to participation.

### 2.2. Procedures

**Figure 1:**
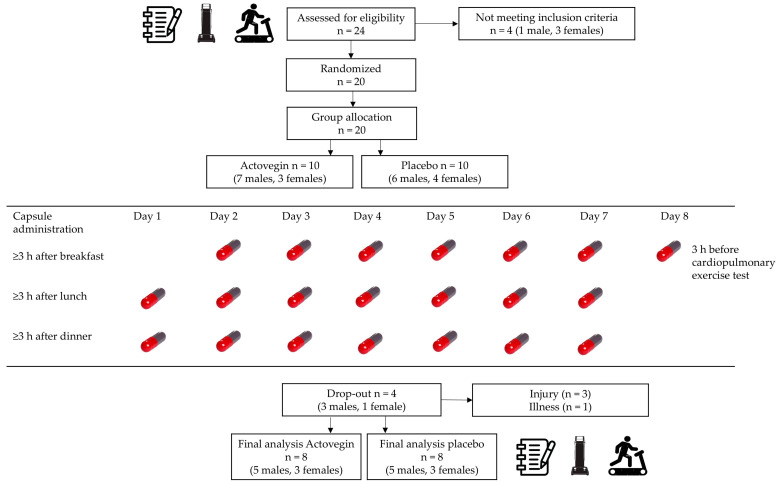
A schematic illustration of the study design and the process adopted for the enrollment, allocation, and drop-out of subjects.

A schematic illustration of the study design and the process adopted for the enrollment, allocation, and drop-out of subjects is shown in Figure 1. A double-blind, placebo-controlled experimental design was adopted whereby 16 randomly assigned subjects completed the placebo (*n* = 8) or Actovegin trials (*n* = 8). During the first visit, all subjects completed screening procedures, had anthropometric measurements taken, and were familiarized with the cardiopulmonary exercise test (verbal instructions and demonstration). Two cardiopulmonary exercise tests were completed, one after familiarization during the screening session and one 7 days following capsule administration. Upon study commencement, all subjects were supplied with a box containing 21 capsules of Actovegin^®^ (Kusum Pharm LLC, Kyiv, Ukraine, packaging of in-bulk manufacturer Takeda Austria GmbH, Linz, Austria) or lactose monohydrate (Fagron NV, Rotterdam, The Netherlands) for the placebo group.

Actovegin was administered orally, since parenteral administration (injections or infusions via intramuscular, intravenous, or intra-arterial routes) are recommended for the treatment of severe clinical manifestations that require a rapid onset of action. Serial oral loading avoids high acute systemic concentrations which may produce side-effects such as immune system complications and allergy symptoms or anaphylactic reactions [8,21]. Also, the oral route [9,12,13,14] of administration was adopted considering the increasing prevalence of abuse in this form across endurance-based sports over the past three decades. Actovegin was administered at a dose of 200 mg (1 capsule) under fasted conditions (≥3 h after eating) three times each day (600 mg·day^−1^) for 7 days of loading. The subjects ingested the last capsule 3 h prior to testing (05:00 to 08:00) to attain peak blood Actovegin concentrations during testing (08:00 to 11:00) [9]. Actovegin dose and time of ingestion were set according to the manufacturer’s instructions. While a 3-week intake period is recommended for the treatment of severe clinical conditions [9], our study adopted a 7-day Actovegin loading considering the good health status of the subjects examined. Also, a 7-day Actovegin loading has been proposed as an effective period for improving skeletal muscle mitochondrial respiration and functional aerobic capacity in a type 1 diabetic male murine model [6]. The same intake procedure was applied in the placebo group, in which the subjects ingested an identical capsule containing lactose monohydrate. The Actovegin and placebo capsules were prepared (in opaque and unidentifiable capsules) and coded by pharmaceutical staff, who had no further involvement in the study, to blind subjects and investigators to the condition being tested. Following data collection, the pharmaceutical staff unveiled the code to the investigators. Also, subjects were instructed not to open the capsule due to substance consistency and time of absorption. Capsule weight varied between the Actovegin (441 ± 7 mg) and placebo capsules (530 ± 7 mg) due to differences in substance density. The weight of the empty capsule was 8 mg, size ‘0’. Capsules were filled in a hand-operated (loading plate 60 holes) filling machine (Aponorm, Hillscheid, Germany).

Throughout the study, subjects received three daily reminders to consume their capsules via a text message using the Viber application. Compliance was registered for each capsule administration if subjects responded to the corresponding text message with a “like”, whereas non-compliance was registered if subjects failed to respond to the corresponding text message. The acceptable compliance rate from each subject for inclusion in the study was ≥80%. Subjects were advised to report any side-effects including allergic reactions (drug-induced fever, shock symptoms), skin and subcutaneous complications (urticaria, flush), headaches, general weakness, dizziness, loss of consciousness, agitation, tremor, stomach discomfort (nausea, vomiting, diarrhea), or other concerns resulting from capsule administration during the study period and to what degree the symptoms had been experienced (with ‘1’ indicating a minimum response and ‘10’ indicating a maximum response). The subjects provided ratings of perceived performance using a 10-point Likert-type scale (with ‘1’ indicating a minimum response and ‘10’ indicating a maximum response) 30 min following completion of the final cardiopulmonary exercise test (item asked: “please rate your overall performance following capsule administration”). Similar Likert-type scales have been widely used in athletes as a reliable [22,23,24] and valid [24,25] indicator of perceived performance. Subjects were instructed to maintain a consistent training routine and diet during the study period, refrain from intense exercise 24 h prior to testing, and avoid the consumption of alcohol and caffeine at least 48 h before testing, which was verbally confirmed with each subject prior to testing.

#### 2.2.1. Body Composition

Upon arrival to each testing session, subjects were weighed to the nearest 0.1 kg unclothed and body composition was also measured (Inbody 770; Biospace Co., Ltd., Seoul, Republic of Korea) between 08:00 and 08:30. The validity (r = 0.90 vs. dual energy X-ray absorptiometry) and reliability (intraclass correlation coefficient [ICC] = 0.98, standard error of measurement [SEM] = 0.91% for males and 0.77% for females) of the InBody 770 in measuring percentage body fat has been supported previously [26].

#### 2.2.2. Cardiopulmonary Exercise Test

The cardiopulmonary exercise test was completed on a motorized treadmill (COSMED T170, Rome, Italy) at a grade of 3% [27]. Test–retest data from our laboratory supported the reliability of VO_2peak_ measurement with this approach (coefficient of variation = 0.4–4.8%, interclass coefficient of variation = 0.91–0.96). All subjects were experienced treadmill users. The test commenced at a speed of 3.5 km·h^−1^, with consistent increases of 0.5 km·h^−1^ each 30 s until exhaustion was reached and subjects could not continue the test. The treadmill protocol (gradient and speed) was selected based on the strong physical fitness of the subjects who had been recruited to ensure the test duration stayed within recommendations of 8–12 min [20,27,28]. For both testing sessions, the Quark metabolic cart with native breath-by-breath technology (COSMED, Rome, Italy) was used to measure all physiological variables including oxygen uptake (VO_2_), respiratory exchange ratio (RER) calculated by dividing carbon dioxide production (VCO_2_) by VO_2_, heart rate (HR), blood pressure, minute ventilation (VE), ventilatory equivalents for carbon dioxide production (VEqVCO_2_), oxygen pulse (VO_2_/HR), and velocity. The metabolic cart was calibrated according to manufacturer instructions prior to each test. Standardized verbal encouragement and instructions were used during testing for all subjects.

HR was continuously monitored via a wireless 12-lead electrocardiogram (COSMED Quark T12x, Rome, Italy). VO_2peak_, RER_peak_, time until exhaustion (Tpeak), HR_peak_, VE_peak_, VEqVCO_2_, VO_2_/HR, and velocity peak were recorded immediately following test completion. HR recovery was registered at the first, second, and third minute following test completion. Blood pressure (Prizma PA1, Kragujevac, Serbia) and rating of perceived exertion (RPE) using Borg’s Category Ratio-10 scale were obtained during the first minute of recovery to check the status of each subject and identify any health risks. Maximal effort was deemed to be achieved by each subject during testing if at least three of the following criteria were met [20,27]: (1) the peak HR attained was within 15 beats·min^−1^ of the individualized age-predicted maximum HR (220—age in years) [29]; (2) voluntary exhaustion was reached as reflected by an RPE of 10; (3) a RER of ≥1.10 was achieved; and (4) a plateau in VO_2_ despite an increase in exercise intensity was evident. Following test completion, first (VT1) and second (VT2) ventilatory thresholds were determined by visual inspection from two experienced investigators with any disagreements resolved via discussion or consultation with a third investigator. The metabolic cart employed an automated program based on the V-slope method where VT1 was found at the intersection of the VCO_2_ vs. VO_2_ graph [30] while VT2 was determined using the VEqVCO_2_ plot where VE increased disproportionately to VCO_2_ [31]. The computerized method was based on procedures reported by Schneider et al. [32], with VT assigned to the intersection of the two straight lines determined by linear regression explaining VCO_2_ versus VO_2_. VO_2_, HR, and velocity were extrapolated at VT1 and VT2. VO_2peak_ was defined as the highest VO_2_ recorded across a 30-s epoch. Standardized guidelines by the American College of Sports Medicine were followed to ensure adherence to appropriate safety measures [20].

### 2.3. Statistical Analyses

An *a priori* power analysis for mixed analyses of variance (ANOVA) containing within- and between-group interactions indicated our study was sufficiently powered given a sample size of 16was recommended using a = 0.05, effect size [ES] = 0.40, and power = 0.80, based on effect magnitudes observed in research [17] examining the dose-dependent effects of Actovegin on oxidative capacity in permeabilized human skeletal muscle (G*power software, version 3.1.9.4; Heinrich Heine University Düsseldorf, Düsseldorf, Germany). Normality of all data was verified using the Shapiro–Wilk test, quantile–quantile (Q–Q) plots, as well as skewness and kurtosis coefficients. Between-group comparisons for subject characteristics at baseline and 7 days later were separately performed with separate independent *t*-tests, whereas a chi-square test was used to analyze sex proportions between groups. Separate 2 × 2 mixed ANOVAs with one within-subjects factor (time) and one between-subjects factor (group) were used to examine the effects of Actovegin on all variables taken during the cardiopulmonary exercise tests. Partial eta-squared ($\eta_{p}^{2}$) was calculated to indicate the ES for each ANOVA and was interpreted as: no effect ($\eta_{p}^{2}$ < 0.04); minimum effect (0.04 < $\eta_{p}^{2}$ < 0.25); moderate effect (0.25 < $\eta_{p}^{2}$ < 0.64); or strong effect ($\eta_{p}^{2}$ > 0.64) [33]. Pairwise comparisons were conducted using Bonferroni post hoc tests when significant effects were observed. A chi-square test was conducted to analyze differences in adverse events following capsule administration, whereas an independent *t*-test was applied to compare perceived performance between groups. Statistical significance was set at *p* < 0.05 with all analyses performed using Statistical Package for the Social Sciences (version 20; IBM Corp., Armonk, NY, USA).

## 3. Results

General characteristics of subjects in each group are presented in Table 1. There were no significant differences in characteristics between the placebo and Actovegin groups. The compliance rate across all subjects was 96% in the Actovegin group (162 out of 168 capsules) and 97% in the placebo group (163 out of 168 capsules).

**Figure 2:**
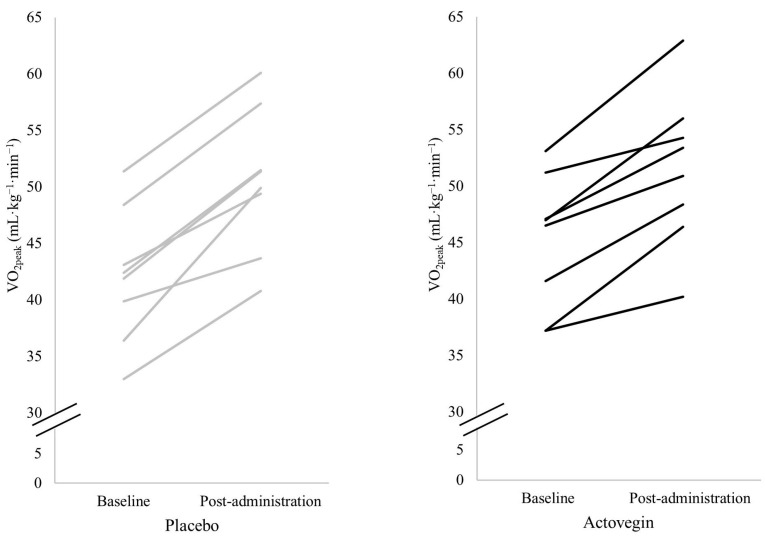
Individual data for peak oxygen uptake (VO_2peak_) in the placebo and Actovegin groups.

The results of the mixed ANOVAs along with the mean ± standard deviation for all variables taken during the cardiopulmonary exercise tests are presented in Table 2. Individual data points for VO_2peak_ across both groups are shown in Figure 2.

The mixed ANOVAs revealed a significant time × group interaction (moderate) only in systolic blood pressure. In turn, post hoc analyses showed that the Actovegin group displayed significantly lower systolic blood pressure post-administration compared to the baseline (*p* = 0.007, $\eta_{p}^{2}$ = 0.416, moderate). Significant main effects for time were observed for VO_2peak_ (strong), RER (strong), VE_peak_ (moderate), VEqVCO_2_ (moderate), oxygen pulse (moderate), VO_2_ at VT1 (moderate), velocity at VT2 (strong), VO_2_ at VT2 (moderate), and peak velocity (moderate). Specifically, both groups displayed significantly lower RER (placebo: *p* < 0.001, $\eta_{p}^{2}$ = 0.611, moderate; Actovegin: *p* = 0.005, $\eta_{p}^{2}$ = 0.438, moderate), alongside significantly higher VO_2peak_ (placebo: *p* < 0.001, $\eta_{p}^{2}$ = 0.843, strong; Actovegin: *p* < 0.001, $\eta_{p}^{2}$ = 0.757, strong), VE_peak_ (placebo: *p* = 0.022, $\eta_{p}^{2}$ = 0.323, moderate; Actovegin: *p* = 0.040, $\eta_{p}^{2}$ = 0.268, moderate), VEqVCO_2_ (placebo: *p* = 0.006, $\eta_{p}^{2}$ = 0.422, moderate; Actovegin: *p* = 0.039, $\eta_{p}^{2}$ = 0.269, moderate), oxygen pulse (placebo: *p* = 0.024, $\eta_{p}^{2}$ = 0.316, moderate; Actovegin: *p* = 0.050, $\eta_{p}^{2}$ = 0.238, minimum), VO_2_ at VT1 (placebo: *p* = 0.006, $\eta_{p}^{2}$ = 0.425, moderate; Actovegin: *p* = 0.039, $\eta_{p}^{2}$ = 0.271, moderate), velocity at VT2 (placebo: *p* < 0.001, $\eta_{p}^{2}$ = 0.994, strong; Actovegin: *p* < 0.001, $\eta_{p}^{2}$ = 0.994, strong), and VO_2_ at VT2 (placebo: *p* < 0.001, $\eta_{p}^{2}$ = 0.572, moderate; Actovegin: *p* = 0.023, $\eta_{p}^{2}$ = 0.318, moderate) post-administration. No significant main effects for group were observed.

No significant differences (*p* = 0.157) were observed between the placebo and Actovegin group in perceived performance during the cardiopulmonary test following capsule administration (placebo: 5.0 ± 3.2, Actovegin: 6.9 ± 1.2). Overall, 3 adverse events in the form of nausea (severity of 3–4 AU) were reported following Actovegin administration with no events reported in the placebo group (between-group comparison, *p* = 0.200).

## 4. Discussion

The results of this study add to the limited physiological and performance data reported in athletes following Actovegin administration. In this regard, we observed parallelled improvements in various physiological and performance variables taken during the maximal cardiopulmonary exercise test across the placebo and Actovegin groups. These findings suggest that although alterations were evident with Actovegin, no further benefits beyond those obtained with a placebo treatment were attained.

The nonsignificant interaction among physiological and performance variables we observed with 7-day Actovegin administration align with data reported by Lee et al. [18] demonstrating no effect of intravenous Actovegin (40 mL) on physiological and performance variables during a maximal, graded arm crank ergometry test in physically active men. On the other hand, the time effects we observed revealed similar benefits to several physiological and performance variables with placebo and Actovegin capsule administration. In this way, when assessing performance-enhancing supplements in placebo-controlled trials, any beneficial effects observed for the treatment of interest should exceed any placebo effects for it to be deemed ergogenic in nature [34]. In turn, as our subjects were athletes engaging in regular exercise and experienced treadmill users, our results are not likely attributable to any learning effects but rather emphasize a potential placebo effect given similar capsule consumption was performed in both groups. These findings lend support for the influence of psychological factors in modulating responses during exercise when capsule administration is involved. Moreover, placebo effects appear to be stronger when subjects believe they are consuming banned substances [35], which may have impacted our study given the associated restrictions on Actovegin use listed in the World Anti-Doping Code. However, future research is encouraged to incorporate a control group that is given no treatment to more clearly understand the precise placebo effects when examining the ergogenic properties of Actovegin [35].

Our findings contrast previous in vitro evidence obtained in permeabilized human skeletal muscle fibers, demonstrating a dose-dependent increase in mitochondrial oxidative phosphorylation capacity, rates of mitochondrial ATP production, and adenosine diphosphate sensitivity of the human skeletal muscle following Actovegin treatment (10 μL·mL^−1^ and 50 μL·mL^−1^) [17]. However, the training status of the sample examined in this previous study could explain these variations in results, since the untrained overweight subjects examined previously held increased potential to improve their exercise capacity, including mitochondrial oxidative function [17]. In addition, discrepancies between our results and those found in clinical settings [8] may also relate to disparities in the conditions tested (e.g., injury vs. performance) as well as route of Actovegin administration (injectable vs. oral). In this regard, the lack of effect in sport settings may relate to the non-clinical conditions and lower dosages of Actovegin consumed orally in our study. Furthermore, despite anecdotal beliefs that Actovegin has similar properties to erythropoietin in enhancing the oxygen-carrying capacity of blood, the absence of any oxygen diffusion-enhancing compound (any peptide, growth factors, or hormone-like substances) in Actovegin may underpin findings observed in our study. 

To determine whether Actovegin represents a risk to the health of athletes, blood pressure was measured following the exercise tests and subjects were advised to report any side-effects experienced. In this regard, we observed a significant decrease in post-exercise systolic blood pressure following the 7-day Actovegin administration compared to baseline, which was not apparent in the placebo condition. Blood pressure comparisons following Actovegin administration are difficult to conduct considering the lack of existing studies in athletes. Nevertheless, our observations align with those previously made in hypertensive patients with metabolic syndrome, demonstrating reduced systolic and diastolic blood pressure following bisoprolol and Actovegin administration in combination during resting [36]. Our findings may relate to the higher post-exercise systolic blood pressure observed at baseline in the Actovegin group (161.9 ± 20.0 mmHg) compared to the placebo group (151.3 ± 21.8 mmHg). In this regard, it has been suggested that the acute responsiveness of blood pressure to exercise may relate to the initial levels observed, with greater reductions experienced in hypertensive subjects compared to normotensive subjects [37]. Although the exact mechanisms underpinning Actovegin in reducing blood pressure is not known, it has been suggested that isolated inositol-phosphate (an ingredient of Actovegin) promotes increased nitric oxide resulting in dilation of the vascular smooth muscle cells to reduce blood pressure [38]. Alterations in post-exercise systolic blood pressure with Actovegin administration were also accompanied by some reporting of mild nausea (*n* = 3), which was not apparent in the placebo condition. While potential side-effects of Actovegin as stated by the manufacturer include stomach discomfort, all adverse events occurred occasionally in each subject who experienced them (2–3 times over 7 days) and were mild in severity. In contrast, Maillo et al. [10] reported an anaphylactic reaction with multi-organ failure in a 22-year-old amateur cyclist after intravenous administration of Actovegin at a dose of 5 mL (40 mg·mL^−1^), probably due to bacterial contamination of the injected site, since the patient’s condition improved using broad-spectrum antibiotics [39]. In other work, no adverse events were reported among subjects when administering Actovegin intravenously at a maximal dose of 40 mL [18]. Consequently, the collective evidence suggests that Actovegin may be mostly well tolerated by athletes with only mild nausea observed in some cases.

Despite the novel findings we observed regarding the effects of orally administered Actovegin on physiological and performance variables during maximal cardiopulmonary exercise in collegiate athletes, the inherent limitations of our study should be acknowledged. First, a control condition not receiving any capsules was not included, which limits our ability to identify inherent placebo effects with capsule consumption. Second, a comprehensive analysis of Actovegin ingredients in the blood was not conducted [7]. Third, although subjects were advised to maintain their eating habits throughout the study, and there was no change in body composition across both groups, food diaries were not collected to better account for the potential confounding influence of nutritional factors on the study results. Fourth, our sample was limited to moderately trained athletes. In turn, the provided results may not be indicative of those found in highly trained, professional athletes since training status may contribute to variations in active substance and placebo-induced improvements in VO_2peak_. Fifth, although adult, moderately trained, collegiate athletes with at least 3 years of training experience in various sports were recruited in our study, differences in the training programs undertaken across athletes may have contributed to the inter-individual variations in responsiveness to capsule administration. In this regard, future research is encouraged to adopt a double-blind, placebo-controlled experimental design in a counterbalanced, crossover manner to account for the potential confounding influence of inter-individual variations on the study results. Sixth, given capsule consumption was checked via a mobile application rather than involving direct administration in-person, our reported compliance is dependent on the accuracy of responses given by participants. Finally, our findings are limited to a general dose of Actovegin at 3 × 200 mg∙day^−1^ (following the manufacturer’s instructions) and relatively low sample sizes in each group (5 males and 3 females) so it is recommended that larger studies examining the effects of Actovegin across various routes of administration and doses should be conducted separately in male and female athletes for more specific evidence to be generated.

## 5. Conclusions

Our study provides important preliminary data regarding the effect of 7-day Actovegin loading at 600 mg∙day^−1^ on the physiological and performance outcomes during maximal cardiorespiratory treadmill exercise. Based on the present findings, a 7-day Actovegin loading at 600 mg∙day^−1^ offers no added physiological or performance benefit beyond a placebo treatment during maximal cardiopulmonary exercise among moderately trained, collegiate athletes. Although Actovegin was relatively well tolerated without severe adverse side-effects, the mild nausea seen in almost half of the subjects taking Actovegin support the need for individualized analyses when conducting nutrition-based experimental trials in applied sport research. Considering the limited number of studies on this topic, the results from the present study may serve as a framework to assist in planning double-blind, placebo-controlled randomized crossover studies with larger sample sizes.

## Figures and Tables

**Table 1:** General characteristics (mean ± standard deviation, mean difference [95% Confidence Intervals (CI)]) of the included subjects in each group.

Characteristic	Placebo Group (*n* = 8)	Actovegin Group (*n* = 8)	Mean Difference (95% CI)	*p*-Value
Weekly training frequency (n)	4.5 ± 1.2	4.4 ± 1.1	0.1 (−1.1 to 1.3)	0.828
Sex, males/females (n)	5/3	5/3		1.000
Age (years)	19.0 ± 0.0	19.3 ± 0.7	−0.3 (−0.8 to 0.3)	0.351
Body height (cm)	180.7 ± 10.1	176.8 ± 11.2	3.9 (−7.6 to 15.4)	0.477
Body mass at baseline (kg)	76.3 ± 15.6	72.9 ± 16.9	3.3 (−14.1 to 20.8)	0.688
Body mass after 7 days (kg)	76.5 ± 15.7	73.0 ± 16.1	3.5 (−13.5 to 20.6)	0.664
Body fat at baseline (%)	19.2 ± 9.3	15.6 ± 6.2	3.7 (5.0 to 12.3)	0.373
Body fat after 7 days (%)	19.0 ± 8.6	15.6 ± 6.7	3.4 (5.0 to 11.7)	0.401
Muscle mass at baseline (%)	45.6 ± 5.9	47.6 ± 4.7	−2.0 (−7.7 to 3.7)	0.462
Muscle mass after 7 days (%)	45.8 ± 5.6	47.7 ± 5.0	−1.9 (−7.6 to 3.8)	0.493
Body mass index at baseline (kg·m^−2^)	23.3 ± 3.9	23.0 ± 2.9	0.3 (−3.4 to 4.0)	0.864
Body mass index after 7 days (kg·m^−2^)	23.4 ± 3.9	23.1 ± 2.8	0.3 (−3.5 to 4.0)	0.887

**Table 2:** Statistical outcomes from the mixed analyses of variance showing time × group interaction, time (baseline vs. post-administration), and group (placebo vs. Actovegin) effects for variables taken from the cardiopulmonary exercise tests.

Variable	Placebo Group (*n* = 8)		Actovegin Group (*n* = 8)		Interaction		Time		Group	
	Baseline	Post-Administration	Baseline	Post-Administration	*p*	$\eta_{p}^{2}$	*p*	$\eta_{p}^{2}$	*p*	$\eta_{p}^{2}$
VO_2peak_ (mL·kg^−1^·min^−1^)	42.1 ± 5.9	50.3 ± 6.4	45.1 ± 6.0	51.6 ± 6.8	0.167	0.132	**<0.001**	**0.893**	0.515	0.031
RER_peak_	1.3 ± 0.1	1.1 ± 0.1	1.3 ± 0.1	1.1 ± 0.1	0.344	0.064	**<0.001**	**0.695**	0.439	0.043
T_peak_ (s)	603.6 ± 100.0	607.3 ± 97.2	606.6 ± 82.5	656.1 ± 88.9	0.212	0.109	0.152	0.141	0.554	0.026
HR_peak_ (beats·min^−1^)	193.3 ± 2.8	193.5 ± 3.9	192.9 ± 9.4	191.4 ± 7.0	0.381	0.055	0.529	0.029	0.686	0.012
HR 1st minute (beats·min^−1^)	168.9 ± 11.8	171.8 ± 3.9	170.8 ± 9.4	171.6 ± 7.9	0.648	0.015	0.397	0.052	0.822	0.004
HR 2nd minute (beats·min^−1^)	149.5 ± 12.7	149.1 ± 8.0	148.4 ± 10.5	147.1 ± 12.0	0.902	0.001	0.819	0.004	0.718	0.010
HR 3rd minute (beats·min^−1^)	135.6 ± 12.9	137.9 ± 7.5	133.4 ± 11.4	132.9 ± 11.7	0.672	0.013	0.787	0.005	0.436	0.044
Systolic pressure (mmHg)	151.3 ± 21.8	151.9 ± 18.3	161.9 ± 20.0	146.3 ± 22.6	**0.036**	**0.278**	**0.050**	**0.247**	0.802	0.005
Diastolic pressure (mmHg)	76.9 ± 7.5	76.9 ± 7.0	78.1 ± 5.9	73.1 ± 7.0	0.373	0.057	0.373	0.057	0.568	0.024
VE_peak_ (L·min^−1^)	120.0 ± 35.1	137.0 ± 30.9	122.1 ± 36.1	136.9 ± 42.6	0.826	0.004	**0.004**	**0.456**	0.957	0.000
VEqVCO_2_	25.4 ± 3.9	28.0 ± 3.7	26.8 ± 5.1	28.7 ± 4.7	0.524	0.030	**0.002**	**0.517**	0.629	0.017
VO_2_/HR (mL·beat^−1^)	20.4 ± 7.6	22.5 ± 7.5	21.1 ± 6.6	22.9 ± 7.2	0.756	0.007	**0.006**	**0.434**	0.877	0.002
HR at VT1 (beats·min^−1^)	167.3 ± 19.2	173.1 ± 19.1	173.9 ± 10.2	172.8 ± 12.7	0.200	0.114	0.377	0.056	0.682	0.012
Velocity at VT1 (km·h^−1^)	8.8 ± 1.8	9.3 ± 2.0	9.9 ± 1.4	9.6 ± 1.2	0.165	0.133	0.811	0.004	0.353	0.062
VO_2_ at VT1 (mL·kg^−1^·min^−1^)	33.5 ± 6.8	38.5 ± 8.0	38.1 ± 6.5	41.7 ± 4.8	0.514	0.031	**0.002**	**0.520**	0.232	0.100
HR at VT2 (beats·min^−1^)	184.3 ± 11.4	188.8 ± 7.7	188.6 ± 9.3	185.4 ± 10.4	0.070	0.216	0.756	0.007	0.913	0.001
Velocity at VT2 (km·h^−1^)	10.9 ± 2.2	11.9 ± 1.7	12.3 ± 1.9	12.8 ± 1.4	0.492	0.034	**<0.001**	**0.997**	0.362	0.060
VO_2_ at VT2 (mL·kg^−1^·min^−1^)	41.1 ± 7.9	49.7 ± 8.2	46.3 ± 8.1	51.4 ± 5.4	0.230	0.101	**<0.001**	**0.628**	0.337	0.066
V_peak_ (km·h^−1^)	12.4 ± 1.6	12.9 ± 1.7	13.0 ± 1.6	13.4 ± 1.5	0.678	0.013	**0.010**	**0.386**	0.491	0.035

Abbreviations: $\eta_{p}^{2}$—partial eta squared; VO_2peak_—peak oxygen uptake; RER—respiratory exchange ratio; T_peak_—time until exhaustion; HR_peak_—peak heart rate attained; HR—heart rate (beats · min^−1^); VE_peak_—peak minute ventilation; VEqVCO_2_—ventilatory equivalents for carbon dioxide production; VO_2_/HR—ratio between oxygen consumption and heart rate, known as the ‘oxygen pulse’; HR at VT1—heart rate at first ventilatory threshold; Velocity at VT1—velocity at first ventilatory threshold; VO_2_ at VT1—oxygen uptake at first ventilatory threshold; HR at VT2—heart rate at second ventilatory threshold; Velocity at VT2—velocity at second ventilatory threshold; VO_2_ at VT2—oxygen uptake at second ventilatory threshold; V_peak_—velocity associated with VO_2peak_; bolded *p*-value and $\eta_{p}^{2}$ indicates a significant effect (*p* < 0.05).

## Data Availability

The raw data supporting the conclusions of this article will be made available by the authors on request.
